# A computational workflow for predicting cancer neo-antigens

**DOI:** 10.6026/97320630018214

**Published:** 2022-03-31

**Authors:** Sandeep Kasaragod, Chinmaya Narayana Kotimoole, Sumrati Gurtoo, Thottethodi Subrahmanya Keshava Prasad, Harsha Gowda, Prashant Kumar Modi

**Affiliations:** 1 Center for Systems Biology and Molecular Medicine, Yenepoya Research Centre, Yenepoya (Deemed to be University), Mangalore, 575018, India; 2 Institute of Bioinformatics, International Technology Park, Bangalore, 560066, India

**Keywords:** Neoantigens, proteogenomics, cancer proteogenomics, multi-omics

## Abstract

Neo-antigens presented on cell surface play a pivotal role in the success of immunotherapies. Peptides derived from mutant proteins are thought to be the primary source of neo-antigens presented on the surface of cancer cells. Mutation data from cancer genome sequencing is often used to predict cancer neo-antigens. However, this strategy is associated with significant false positives as many coding mutations may not be expressed at the protein level. Hence, we describe a computational workflow to integrate genomic and proteomic data to predictpotential neo-antigens.

## Background:

Cancer is the second leading cause of morbidity and mortality worldwide. As per a survey conducted by Cancer Research UK in 2018, there are 17 million new cases worldwide, and cancer-related death has risen to 9.6 million [1]. Cancer is heterogeneous. As cancer cells proliferate, they create tumors with genetically heterogeneous cells making it challenging to treat [2], [3]. Chemotherapy and targeted therapies are effective only in select cancer types. The advent of immunotherapy has revolutionized cancer treatment in the last decade [4]. For example, checkpoint blockade-based treatments have significantly improved cancer survival [5,6]. Other immunotherapy treatments such as adoptive cell transfer therapy and small molecule inhibitors are widely used to treat various cancer types [7,8]. The success of immunotherapy strategies is dependent on presentation of neo-antigens on cancer cell surface. These neo-antigens are presented by MHC complex on the cell surface, which are recognized by T cells [9]. Cancer genome sequencing has revealed thousands of mutations associated with various cancers [10,11,12]. Mutation data from cancer genome sequencing is often used to predict cancer neo-antigens. However, this approach can result in false positives as many mutations may not be expressed at the protein level [13] [14]. We previously developed a computational workflow to integrate genomic and proteomic data to identify coding variations [15]. Therefore, we describe a workflow to predict cancer neo-antigens.

## Methods:

### Genomics and proteomics data analysis:

Genomics datasets [18][19][20][21] were analyzed using the CusVarDB tool. A custom protein database was developed by incorporating coding mutations that was used to carry out proteomics searches. Proteomeics searches were carried out using Proteome Discoverer 2.3 (Thermo Fisher Scientific, Bremen, and Germany). The cancer type-specific raw files were searched against the corresponding customized variant protein database using Sequest-HT search engine [22]. The search parameters were set as reported in the original studies [23], [24] [25]. False discovery rate (FDR) was set to 1% at PSM, peptide, and protein levels. (Figure 1-a - see PDF) describes the proteogenomics workflow used in our study.

### Workflow development:

The workflow is created using snakemake version 6.12.3 [16]. All the supporting scripts for the workflow are written in Python 3.9. This snakemake workflow requires variant annotation results from ANNOVAR [17] and proteomics search results. Proteomics data is searched against a custom database that incorporates coding mutations identified in genomics data. Peptides that do not have sequence variations are filtered by mathching sequences to reference protein sequence database. Variant peptides are assigned unique accessions and are provided as a tab-delimited or comma-separated file that can be queried using SQL.

### Prediction of neoantigens:

Neoantigen prediction was performed using offline version of net MHCpan 4.1 [26]. We kept a window of ± 15 amino acid sequence from the variant amino acid. It created an overall sequence length of 30 amino acids. These sequences were stored in FASTA format to perform predictions. HLA allele information for corresponding cell lines was taken from the literature [27][28], and Expasy (https://web.expasy.org/cellosaurus/).

### Code availability:

Workflow is available at Github (https://github.com/sandeepkasaragod/Proteogenomics_workflow).

## Results and Discussion:

Our study was carried out using datasets from twenty-five cancer cell lines. Fourteen datasets were from TNBC, five from ovarian cancer, and six from colon cancer. The exome datasets were subjected to variant analysis. We identified 125,687 unique non-synonymous variants from 25 datasets. Non-synonymous variants were incorporated into protein sequences from RefSeq database to create a custom variant protein database to perform proteomics data analysis. Proteomics searches identified 231,886 unique peptides from 25 datasets. Overall, we identified 4,673 variant peptides corresponding to 1,249 genes (Figure 1b - see PDF). We also identified 1,297 variants that correspond to 295 genes reported in COSMIC [29] and ClinVar [30]. These include well-known cancer-related genes such as TP53, KRAS, EGFR, AARS, ACTN4, SAMHD1, and many other genes. Enrichment analysis showed genes involved in important functions including cell division, cellular metabolic process, cellular localization, and other events. (Figure 1d - see PDF). The same set of peptides was run on Net MHCpan for neoantigen prediction. We identified a total of 5,865 neoantigens with strong binding affinity. We also identified corresponding wild type peptides for 1,915 variant peptides. We predicted binding affinity for corresponding wild type peptides. Of these, 707 variant peptides had a stronger binding affinity when compared to their wild type (supplementary available at GitHub). Cluster Profiler analysis of these variant proteins showed their involvement in various cancers including breast cancer, colorectal adenocarcinoma and cervical cancer (Figure 1c - see PDF). In this study, we utilized the power of multi-omics datasets to predict potential cancer neo-antigens. We developed a proteogenomics data analysis workflow using snakemake package. The workflow is highly customizable and efficiently executed in a condo environment.

## Conclusions:

Identification of cancer neoantigens is important to develop effective immunotherapy strategies. Predicting cancer neoantigens using genomic data alone can result in several false positives. In this study, we present an integrated approach combining genomic and proteomic data to predict cancer neoantigens. This computational workflow can be used on any dataset where both genomic and proteomic data is available.

## References

[1] Bray F (2018). CA Cancer J Clin..

[2] Bedard PL (2013). Nature..

[3] Pucci C (2019). Ecancermedical science..

[4] Han X-J (2020). Front Cell Dev Biol..

[5] Sharma P, Allison JP (2015). Science.

[6] Brahmer JR (2012). N Engl J Med..

[7] Efremova M (2017). Front Immunol..

[8] Wu W (2020). Oncol Lett..

[9] Roudko V (2020). Front Immunol..

[10] https://pubmed.ncbi.nlm.nih.gov/32025007/.

[11] Alexandrov LB (2020). Nature..

[12] Yuan Y (2020). Nat Genet..

[13] Geiger T (2010). PLoS Genet..

[14] Zhang B (2014). Nature..

[15] Kasaragod S (2020). F1000Res..

[16] Molder F (2021). F1000Res..

[17] Wang K (2010). Nucleic Acids Res..

[18] Abaan OD (2013). Cancer Res..

[19] Daemen A (2013). Genome Biol..

[20] Barretina J (2012). Nature..

[21] Ghandi M (2019). Nature..

[22] Eng JK (2008). J Proteome Res..

[23] Lawrence RT (2015). Cell Rep..

[24] Yen TY (2017). J Proteome Res..

[25] Gholami AM (2013). Cell Rep..

[26] Reynisson B (2020). Nucleic Acids Res..

[27] Adams S (2005). J Transl Med..

[28] Robinson J (2020). Nucleic Acids Res..

[29] Tate JG (2019). Nucleic Acids Res..

[30] Landrum MJ (2018). Nucleic Acids Res..

